# Male Mating Competitiveness of a *Wolbachia*-Introgressed *Aedes polynesiensis* Strain under Semi-Field Conditions

**DOI:** 10.1371/journal.pntd.0001271

**Published:** 2011-08-02

**Authors:** Eric W. Chambers, Limb Hapairai, Bethany A. Peel, Hervé Bossin, Stephen L. Dobson

**Affiliations:** 1 Department of Entomology, University of Kentucky, Lexington, Kentucky, United States of America; 2 Institut Louis Malardé, Papeete, French Polynesia; Yale School of Public Health, United States of America

## Abstract

**Background:**

Lymphatic filariasis (LF), a global public health problem affecting approximately 120 million people worldwide, is a leading cause of disability in the developing world including the South Pacific. Despite decades of ongoing mass drug administration (MDA) in the region, some island nations have not yet achieved the threshold levels of microfilaremia established by the World Health Organization for eliminating transmission. Previously, the generation of a novel *Aedes polynesiensis* strain (CP) infected with an exogenous type of *Wolbachia* has been described. The CP mosquito is cytoplasmically incompatible (i.e., effectively sterile) when mated with wildtype mosquitoes, and a strategy was proposed for the control of *A. polynesiensis* populations by repeated, inundative releases of CP males to disrupt fertility of wild females. Such a strategy could lead to suppression of the vector population and subsequently lead to a reduction in the transmission of filarial worms.

**Methodology/Principal Findings:**

CP males and F1 male offspring from wild-caught *A. polynesiensis* females exhibit near equal mating competitiveness with F1 females under semi-field conditions.

**Conclusions/Significance:**

While laboratory experiments are important, prior projects have demonstrated the need for additional testing under semi-field conditions in order to recognize problems before field implementation. The results reported here from semi-field experiments encourage forward progression toward small-scale field releases.

## Author Summary

*Aedes polynesiensis* is the primary mosquito vector of lymphatic filariasis (LF) in the island nations of the South Pacific. Control of LF in this region of the world is difficult due to the unique biology of the mosquito vector. A proposed method to control LF in the Pacific is through the release of male mosquitoes that are effectively sterile. In order for this approach to be successful, it is critical that the modified male mosquitoes be able to compete with wild type male mosquitoes for female mates. In this study the authors examined the mating competitiveness of modified males under semi-field conditions. Modified males were released into field cages holding field-collected, virgin females and field collected wild type males. The resulting proportion of eggs that hatched was inversely related to the number of modified males released into the cage, which is consistent with the hypothesized competitiveness of modified males against indigenous males. The outcome indicates that mass release of modified *A. polynesiensis* mosquitoes could result in the suppression of *A. polynesiensis* populations and supports the continued development of applied strategies for suppression of this important disease vector.

## Introduction

Lymphatic filariasis (LF) is a mosquito-borne disease that can lead to gross disfigurement (lymphedema and elephantiasis) and disability. In addition to the severe pain that often accompanies LF, many affected individuals suffer psychological distress due to associated social stigmas. In severe cases, individuals may become physically incapacitated [1]. Thus, filariasis can place a significant socioeconomic burden on individuals, communities, and healthcare systems [2].

Because there is currently no vaccine available for LF, current control of this disease is based on the regular administration of anti-filarial compounds to the entire at-risk population. Although the drugs do not kill adult filarial worms, they are microfilaricidal and decrease the level of infectious larvae in the blood. In theory, mass drug administration (MDA) campaigns that last longer than the fecund life span of adult filarial worms, or approximately five years [3], should eliminate LF altogether. However, experience has shown that the strategy can be complicated within some systems [4].

The South Pacific region has a longstanding history of public health campaigns directed toward the control of filarial transmission, including some areas that have practiced mass drug administration since the 1950′s [5]. In the case of Maupiti, a small, relatively isolated island in French Polynesia, low-level transmission persists despite more than three decades of MDA [6], suggesting that MDA alone may be inadequate for the elimination of LF in some areas. In such cases, integration of complementary vector control strategies may be required [4].

Throughout much of the South Pacific, the primary vector of human filariasis is *Aedes polynesiensis*, a mosquito that exhibits higher transmission efficiency when microfilaremia is low [7], [8]. This pattern of negative density-dependent transmission has been hypothesized to contribute to the inability of MDA to eliminate LF in some regions of the Pacific. Control of *A. polynesiensis* is difficult because the mosquito is exophilic and breeds in both artificial containers and natural sites, such as tree holes, crab burrows, shells, and leaves [9], [10]. Multiple attempts have been made to control *A. polynesiensis*, using a variety of measures, including the competitive replacement of *A. polynesiensis* with a refractory species *A. albopictus* on the atoll of Taiaro [11]. Additional control strategies based on the manipulation of vector breeding site have utilized polyester beads, larvivorous fish *Gambusia affinis* and *Poecilia reticulata*, and the copepod *Mesocyclops* [12] as well as land-crab burrows baits [10], [13], [14]. These efforts have been met with limited success [4], [10], [15] as the wide range and number of available breeding sites, coupled with the often rugged and inaccessible terrain of South Pacific island nations, makes it unfeasible to sustain vector control strategies across the numerous, widely dispersed islands.

An additional strategy for *A. polynesiensis* control in the Pacific is a variation of Sterile Insect Technique (SIT). SIT is based upon the release of sterile males in order to suppress and eliminate an insect species. A frequently noted example is sterile male releases used to eliminate the screwworm fly, *Cochliomyia hominovorax*, from the United States, Mexico, Central America and Curacao in the 1950′s [16], [17], [18]. Weekly releases of 40 million sterile males are ongoing in Panama, to prevent the reinvasion of *C. homnivorax* from South America [19].

Success with the screwworm encouraged research into the broader use of SIT in additional insects of both economic and medical importance. The technique has been successfully employed in the eradication of the melon fly, *Bactrocera cucurbitae*, from Southwestern Japan [20], [21] as well as for the eradication the tsetse fly, *Glossina austeni*, from Zanzibar [22]. SIT is also a critical component in controlling and eliminating the Mediterranean fruit fly, *Ceratitis capitata* (Wiedemann), from California [23].

The earliest attempt at employing SIT for mosquito control was made between 1959-1961, when the USDA used ionizing radiation to sterilize *Anopheles quadrimaculatus* pupae [24]. Subsequent attempts by a variety of researchers involved irradiation of pupae from *Aedes aegypti* [25], *Culex quinquefasciatus* [26], [27], and *Culex tarsalis* [28]. These attempts were met with limited success, in part because the process of irradiation affected the male fitness in terms of locating and mating with wild females [29]. Recent efforts at radiation-based SIT of mosquitoes is focused on the release of sterilized *Anopheles arabiensis* in the Sudan [30], [31] as well as on the irradiation of *Aedes albopictus* in Italy [32].

As an alternative to irradiation, several control programs based on chemical sterilization of male *A. aegypti*, *Cx. quinquefasciatus*, and *Anopheles albimanus* were initiated in the 1970′s and 1980′s [33], [34], [35], [36]. However, these have not been extended, primarily due in part to environmental concerns associated with residual chemosterilants on the released mosquitoes [35].

Incompatible Insect Technique (IIT) is similar to SIT, but IIT relies upon embryonic lethality resulting from cytoplasmic incompatibility (CI) induced by the maternally transmitted intracellular bacterium *Wolbachia pipientis* [37]. *Wolbachia* is a naturally occurring endosymbiont of arthropods that renders mosquitoes reproductively incompatible when mated to individuals with a differing infection type [38]. Because it does not rely on modifying the males through irradiation or chemical treatment it may avoid some of the male fitness problems associated with SIT programs in the past [29]. The IIT approach provides a relatively rare example of a successful mosquito field trial, when Laven used this technique to successfully eliminate *Culex pipiens fatigans* from a region of Burma in 1967 [39].

In 2008, Brelsfoard *et al* proposed a vector control strategy for the South Pacific that is based on IIT [37]. Field surveys to date have shown that natural populations of *A. polynesiensis* are infected with a single *Wolbachia* type [40], [41], [42]. An artificially infected *A. polynesiensis* strain (CP) was generated by introgressing a *Wolbachia* type from *Aedes riversi* into the *A. polynesiensis* genotype. Laboratory tests demonstrated that the CP strain was bidirectionally incompatible with naturally infected mosquitoes. Subsequent tests also demonstrated that CP and wild type males exhibited near equal mating competitiveness under laboratory conditions [43].

Previous SIT programs have repeatedly demonstrated the importance of confirming laboratory results within field conditions prior to large scale implementation [44]. Specifically, laboratory strains typically have lower relative fitness compared to wild type mosquitoes, and the difference in fitness may not become apparent until the mosquitoes are moved from the stable environment of the laboratory and placed under more natural conditions. Here, we describe male mating competitiveness assays between CP and wild-type males, performed under semi-field cage conditions.

## Methods

### Insect maintenance and strains

Two mosquito strains were compared in this study: the bidirectionally incompatible CP strain [43], which has been maintained in the laboratory for over twenty generations and the wild-type *A. polynesiensis* Atimaono strain (APA). APA was collected from a coconut grove in Atimaono, Tahiti (17°46′41.44"S 149°27′14.23"W). In order to minimize the effects of laboratory maintenance, eggs from field-collected APA females were reared. The resulting F1 APA adults were used for experiments.

To minimize differences caused by immature rearing conditions, CP and APA were reared under identical laboratory conditions. Larvae were maintained on a 60 g/L liver powder solution (MP Biomedicals LLC, Solon, OH). Adult mosquitoes were maintained on 10% sucrose. Ambient temperature ranged from 23–31°C. Relative humidity was maintained at or above 80% using a humidifier.

### Semi-field cage design

Field cages were 223×127×102 cm tents (Aura, Marmot Mountain LLC, USA) placed on a platform with its legs in a water moat were used to prevent ants from entering the field cages. Each field cages was covered by a 365×275×215 cm screen house (Ozark Trail, Model WMT-1290S, USA). The two-cage design was employed in order to reduce the potential for accidental escape of laboratory-reared mosquitoes or the accidental introduction of wild mosquitoes. Mosquitoes observed inside the external screen house were killed before opening the inner cage. A 3×5 m tarpaulin was suspended over each field cage, to provide shading and protection of cages (e.g., during periodic heavy rainfall). Each field cage contained a black plastic flowerpot as a resting area and containers of a 10% sucrose solution as a carbohydrate resource. A Hobo data logger (U12-012, Onset Computer Corp., USA,) was placed within cages to record temperature, relative humidity and light intensity. Rainfall was monitored using a rain gauge located within 500 meters of the cages.

The field study was conducted on the campus of the Institut Louis Malardé, Paea-Tahiti, French Polynesia. Field cages 1–3 were placed under a *Hibiscus tiliaceus* canopy, while field cages 4–6 were surrounded by *Wedelia trilobata*, *Spathodea campanulata*, *Citrus*, *Musa*, and *Acacia*. The natural vegetation provided protection from direct sunlight and heat. However, some of the surrounding plants, especially the *W. trilobata* growing underneath and the vines growing directly on the platforms, were trimmed periodically to prevent ants from accessing the cages.

### Rearing and release

In order to ensure virginity of adult mosquitoes, pupae from each strain were individualized into 10 ml tubes with water and allowed to eclose. Mosquitoes were sexed at the adult stage. Individuals were then released into 30.5×30.5×30.5 cm cages (Cat. No. 1450; Bioquip Corp., USA). Males and females were held in separate cages in the laboratory until sexually mature. At the time of release, males were approximately 48 hours post-eclosion, and females were approximately 24 hours post-eclosion. Fifty virgin APA females and fifty virgin males were released into cages.

For the mating competitiveness trials, two experimental designs were performed. The first design (Experiment A) compared three APA:CP male ratios (0∶50, 25∶25, 50∶0). Experiment A was performed on two different days and each treatment was repeated in two different tents on each of those days (4 treatment replicates). The second design (Experiment B) compared five APA:CP male ratios (0∶50, 12∶38, 25∶25, 38∶12, 50∶0). Experiment B was performed on three different days and each treatment was represented once on each of those days (3 treatment replicates). In both experiments the female:male sex ratio was 50∶50. For both designs, the different treatments were randomly assigned to different cages, to avoid a potential bias due to environmental variation between cage locations. Males were released into field cages first, followed by virgin females.

### Recapture and egg monitoring

Twenty-four hours after releasing mosquitoes into field cages surviving mosquitoes were removed from cages using a backpack aspirator (Model 1412, John Hock Co., USA), and male and female mortality was recorded. Males were separated from females to avoid subsequent mating events, and both sexes were placed into separate Bioquip cages as described above and held in the insectary.

Female mosquitoes were blood fed on laboratory mice (*Mus musculus*) at the Institut Louis Malardé (Tahiti, FP), with the authorization of the “Commission permanente de l'assemblée de la Polynésie Française (Tahiti)” [Deliberation N°2001-16/APF] and in accordance with French regulations. Engorged mosquitoes were individualized into oviposition cups and provided with a sugar source. Following embryonation, eggs were hatched by flooding and placed under a vacuum for one hour. Hatch rates were determined by examining eggs using a Leica EZ4D dissecting microscope (Leica Microsystems GmbH).

Females that produced egg batches resulting in less than ten larvae were dissected in Ringer Lactate B. Braun buffer (B. Braun Medical SA, Spain), and the insemination status was determined by direct visualization of sperm in the spermathecae under 60x magnification using a Leica Diaplan compound light microscope (Leica Microsystems GmbH, Germany). In addition to qualitative insemination status, the number of inseminated spermathecae was also recorded. Broods in which more than 10% of the eggs produced larvae were considered to be from a compatible cross.

### Data analysis

Male mortality and egg hatch data were arcsine transformed, and Analysis of Variance (ANOVA) was used to compare male mortality for the different treatments. Male mating competitiveness was analyzed using a Chi-square goodness of fit test to compare observed and expected numbers of hatching broods for each APA:CP male ratio. An additional estimate of CP male competitiveness was determined using the method of Fried [45]. Briefly, this statistic is derived through the equation
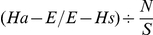



Where Ha  =  the % egg hatch of normal (N) males x normal females, E  =  the % egg hatch of a mixed ratio of normal and sterile males, and Hs  =  the % egg hatch of sterile (S) males x normal females.

Egg batches laid by non-inseminated females were excluded from the analysis. Egg hatch data was compared using the Kruskal-Wallis test. Pairwise comparisons for egg hatch between treatments were performed using Wilcoxon Rank Sum test with Bonferroni correction. All statistical tests were performed using JMP 8.0.1 (SAS Institute, Cary, NC).

## Results

This study was conducted over the course of 3 months (April-June) in 2009 on the island of Tahiti, French Polynesia. Temperatures during the course of the experiments ranged from 21–33°C. The percent relative humidity ranged from 64–97%. Precipitation levels were negligible during the study except for the final replicate of experiment B, when 29.3 mm of precipitation was recorded.

The mean 24-hour mortality by cage treatment for male mosquitoes in Experiment A and experiment B is shown in Table 1. There was no significant difference in male mosquito mortality between the three treatments in experiment A (ANOVA; *P*>0.05) or between the five treatments in experiment B (ANOVA; *P*>0.05). The pooled mean 24-hour male mortality for experiment A was 7.7%±0.5 % (SEM) while the pooled mean 24-hour mortality for males in Experiment B was 7.1%±0.3% (SEM).

**Table 1:** Mean 24-hour male mortality in *A. polynesiensis* mosquitoes.

Experiment	Ratio APA:CP	% mortality ± SE
A	0∶50	9.0±1.7
	25∶25	5.0±2.4
	50∶0	9.0±4.4
B	0∶50	6.7±3.5
	12∶38	8.0±4.2
	25∶25	5.3±1.3
	38∶12	7.3±1.8
	50∶0	8.0±4.0

**Figure 1:**
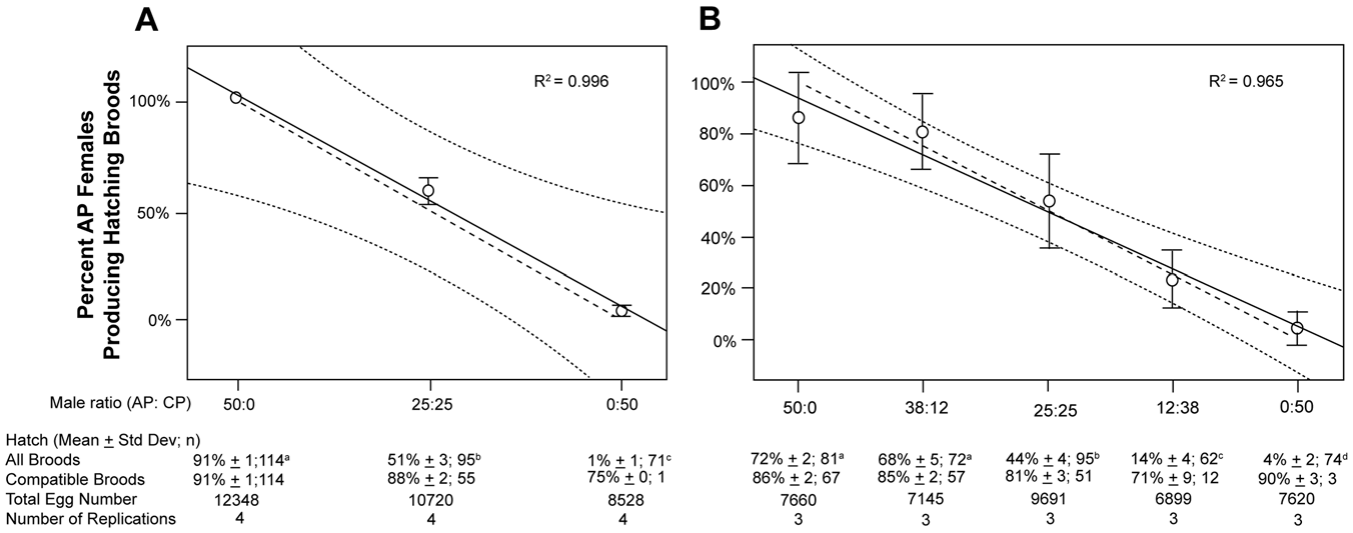
Assessment of *A. polynesiensis* CP male competitiveness in field cages. A; The results of Experiment A. B; The results of Experiment B. Females were considered to have produced a hatching brood when egg hatch was >10%. Circles and bars indicate the mean ± standard deviation for each male ratio. The trend line (dashed line) with 95% confidence intervals (dotted line) is generated based on observed values. Predicted values of compatibly mated broods (solid line) were calculated assuming equal competitiveness of the APA and CP males. R^2^ value is fitted to the observed values. Females were scored according to the observed egg hatch rate as either ‘compatible mating’ (>10% egg hatch) or ‘incompatible mating’ (<10% egg hatch). ‘All broods’ is the average egg hatch resulting from both compatible and incompatible broods. The egg hatch rates are based upon combined oviposition of females within the same treatment field cages. Differing superscripted letters indicate significant differences. Experiment A (Wilcoxon Rank-Sum test, p<0.016, Bonferroni corrected); Experiment B (Wilxocon Rank-Sum test, p<0.01, Bonferroni corrected).

Assuming equal mating competitiveness, one would expect the proportion of females mating with CP males, and therefore producing inviable egg broods, to equal the proportion of CP males present. Figure 1 illustrates that no significant difference was observed between the expected and observed number of hatching broods for any of the treatments in Experiment A (Chi-square; P>0.75). The observed brood hatch rate decreased from 91% to 1%, inversely proportional to the number of CP males present (R^2^>0.99). Again in Experiment B, no significant difference was observed between the expected and observed brood hatch rates (Chi-square; P>0.10; Figure 1B). The observed brood hatch rate decreased from 72% to 4%, inversely proportional to the number of CP males (R^2^ = 0.96). The mean competitiveness value (C) for CP mosquitoes was calculated for both experiments, at 0.84±0.04 and 0.92±0.48 for Experiments A and B, respectively.

**Figure 2:**
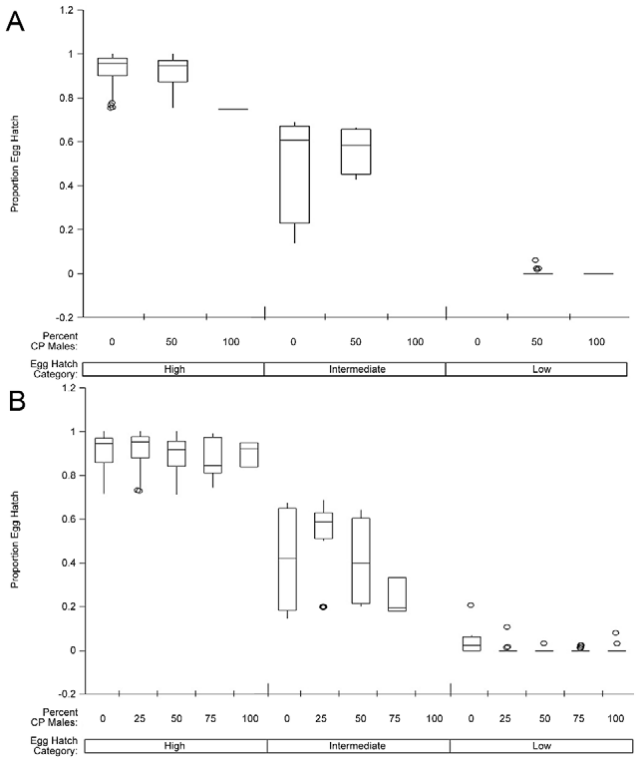
Box-plot showing the distribution of brood hatch rates for treatment groups from Experiment A and Experiment B. A; The results from experiment A. B; The results from experiment B. Median and 1^st^ (Q_1_) and 3^rd^ (Q_3_) quartiles of brood hatch rates for each of the CP:APA treatments. The whiskers of the box-plot represent 1.5*Q_1_ and 1.5*Q_3_ and circles represent outliers. Hatch categories; h = high (≥70%), i = intermediate (11–69%), l = low (≤10%).

Broods were considered compatible when hatch rates were greater than 10%. No significant difference was observed in egg hatch rates from compatible broods (i.e., hatching) of the three treatment groups from Experiment A (Figure 1A; P = 0.07) nor from the five treatment groups from Experiment B (Figure 1B; P = 0.18). Compatible broods were further stratified into two groups; those with an intermediate hatch rate (11%–69%) and those with a high hatch rate (>70%). There was no significant difference observed in egg hatch rates for broods from the 5 treatments in the intermediate category from experiment B (Figure 2; P = 0.16) nor was a significant difference observed in egg hatch rate for broods from the 5 treatments in the high category (Figure 2; P = 0.12). There was also no difference between the 5 treatments in experiment B in the number of hatching broods in the intermediate category (χ^2^ = 3.62; P = 0.46).

In order to confirm that inviable broods were due to CI and not to a failure of the females to mate, all females that produced incompatible brood broods were dissected, and their insemination status was determined. Of the examined females, five of 610 were unfertilized (Table 2). These females were excluded from the analyses. The majority of dissected females (96.1%) had two spermathecae inseminated. The remaining females were observed to have sperm present in a single spermathecum (1.8%) or all three spermathecae (1.3%).

**Table 2:** Insemination rate of *A. polynesiensis* females after 24 h in field cages.

	Number of spermathecae inseminated			
	0	1	2	3
Total	5	11	586	8
Percent	0.8	1.8	96.1	1.3

## Discussion

One of the factors determining the success of an IIT vector control strategy will be the ability of the released males to compete with indigenous males. Colonization and extended maintenance in the insectary can select for inappropriate mating behaviors adapted to the unnatural conditions found in the insectary (e.g. cage size, lighting, temperature, humidity). For example, while wild *Anopheles* form mating swarms at dusk, their laboratory counterparts may be forced to swarm in the dark [46]. The resulting released males that attempt to mate in the dark would be unlikely to find mates. An additional example is provided by a control program focused on the release of *Culex tritaeniorhynchus*, where mating behaviors were selected which resulted in assortative mating in the field [47]. Extended colonization may also allow for the masking of mating barriers that exist between release males and females found in the wild [48].

Therefore, it is critical to perform intermediate tests under semi-field conditions to identify potential problems before proceeding to field implementation. Here, we report that CP males are sexually (but not reproductively) compatible with field collected *A. polynesiensis* females and that under semi-field conditions, CP males exhibit mating competitiveness that is indistinguishable from field collected *A. polynesiensis* males.

Based on male competitiveness estimates (C) of 0.84 and 0.92 for experiments A and B respectively it is estimated that the number of CP males released in any control program would need to be increased by 1.25 to 1.3 times the number that would be needed if CP males had a competitiveness value of 1. This compares favorably with the estimated (C) of 0.785 reported for the MACHO strain of *Anopheles albimanus* in field tests assessing male mating competitiveness in El Salvador in the 1970s [49].

APA females are inseminated at equal rates by both CP and APA males, as less than 1% of the examined APA females had no inseminated spermathecae. The predominance of females with two inseminated spermathecae (96%) is consistent with a prior report [50]. This is additional evidence that the low brood hatch rates observed in treatment cages with increasing ratios of CP:APA males is due to CI and not due to the lack of successful matings of APA females with CP males.

Within the anophelines there is evidence for multiple insemination of female mosquitoes [51], [52] and previous reports indicated that *Aedes aegypti* is typically inseminated only once [53]. The results from this study also support the hypothesis that female *A. polynesiensis* only utilize sperm from a single mating. A similar finding was observed in laboratory studies evaluating the mating competitiveness of CP mosquitoes with laboratory strains of *A. polynesiensis* [37]. Although this study does not preclude the possibility that females are mating with more than one male, the lack of a reduction in egg hatch rate among treatments with mixed ratios of CP and APA males points to preferential utilization of a single inseminated spermathecae.

Females exposed only to incompatible CP males produced 145 broods, of which four produced an egg hatch greater than 10%. A potential explanation is that one or more wild *A. polynesiensis* males were accidentally introduced into the field cage as researchers entered the cage. Wild type males were commonly noted in close proximity to field cages. Additional explanations include the inadvertent introduction of either female CP mosquitoes (via a failure to completely separate females from males) or gravid wild *A. polynesiensis* mosquitoes into the experimental field cage. Close examination of the four hatching broods reveals that three were collected from the same field cage replicate in Experiment B. The hatch rate resulting in these three broods was >80%, which is most congruent with accidental entry of a wild type male. The remaining example occurred in Experiment A, and the hatch rate was 75%, which is congruent with the accidental introduction of a CP or previously-inseminated female. It is emphasized that broods with hatch rates <10% were considered to be from incompatible matings. CI does not necessarily equate with perfect sterility, as the strength of cytoplasmic incompatibility can be affected by the host species as well as by the strain of Wolbachia involved [54].

Although this study demonstrates that CP males are highly competitive with the APA field strain males, it should be noted that the true effectiveness of a release program is based upon a number of factors beyond just that of male competitiveness. Release program effectiveness can be impacted by the frequency and distribution of male releases, the ability of released males to locate mates, and the longevity of released males within the field environment [29]. The latter will require open field releases. The results of this study support the progression to small-scale field releases to test the efficacy of incompatible CP male releases as a vector control strategy for the South Pacific.
